# Completing the *Candida* Loop

**DOI:** 10.1371/journal.pbio.0050270

**Published:** 2007-09-18

**Authors:** Kira O'Day

Imagine identical twin sisters, one short, dark-haired, and zaftig; the other tall, blond, and willowy. You’re not likely to run across this pair, since identical twins possess matching genomes and thus, when raised under the same conditions, look alike. But some organisms, like the fungus Candida albicans, can assume dramatically different, heritable forms even though they share identical genes. A common human pathogen, C. albicans, most commonly exists in what is known as the white form—rounded yeast cells that grow as hemispheric white colonies. But in rare cases, it spontaneously switches to a less-stable opaque form, with elongated cells and flat, grayish colonies. The switch to the opaque form not only changes the fungus’s appearance, but more importantly permits C. albicans cells to interact differently, both with each other and with their mammalian hosts. White cells fare better when infecting the bloodstream, for example, while opaque cells are more optimized for colonizing skin.

Previous research has shown that two genes, white-opaque regulator 1 (*WOR1*) and enhanced filamentous growth 1 (*EFG1*), are involved in white-opaque switching. In a new study, Rebecca Zordan, Mathew Miller, and colleagues fleshed out the circuitry responsible for the C. albicans white-opaque switch, identifying two genes that, along with *EFG1* and *WOR1*, make up a network of positive-feedback loops. To do this, they started with over 400 genes that are expressed differently in white and opaque cells. They eventually focused on two genes, one that they named *WOR2* and one called *CZF1*, which had been previously studied in other processes in C. albicans. Both of these genes show increased expression in opaque cells and, like *WOR1* and *EFG1*, code for transcription factors (proteins that, by binding to DNA, regulate when, where, and how much RNA is synthesized from specific genes).

To determine whether *WOR2* and *CZF1* are involved in regulating the white-opaque switch, the authors created C. albicans mutants in which either *WOR2* or *CZF1* was deleted. Deletion of either of these genes dramatically reduced the rate of white-opaque switching, indicating that, in nonmutant cells, they function as switch activators. When an extra dose of *Czf1* was introduced into nonmutant white cells (a technique known as ectopic expression), nearly all of the cells switched to the opaque form. This was not the case when *WOR2* was ectopically expressed, indicating that additional *Wor2* is not sufficient to activate switching.

**Figure:**
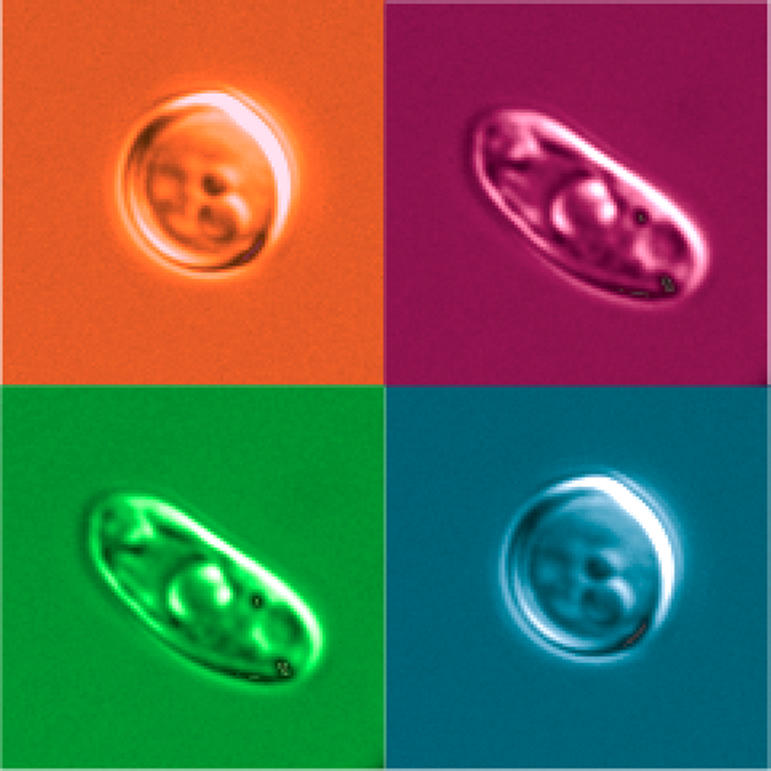
Images of round white-phase and elongated opaque-phase Candida albicans cells. (Differential interference contrast microscopy images, pseudocolored.)

Next, the authors wanted to find out how *CZF1* and *WOR2* interact with *WOR1* and *EFG1*, the previously identified genes, to regulate switching. Using a series of mutants with either one or two genes deleted (along with ectopic expression), the authors deduced that *WOR1* regulated both *WOR2* and *CZF1*, since ectopic expression of *WOR1* caused increased white-opaque switching even when *WOR2* or *CZF1* were deleted. It was known that *EFG1* is required to stably maintain the white state from one generation to the next. The authors now propose that *EFG1* is repressed by *CZF1* in opaque cells.

To test whether the *WOR1* protein directly interacts with the DNA of the other three genes in opaque cells, the authors performed a series of experiments (called chromatin immunoprecipitation assays) to determine whether a given protein binds to or is localized to a specific DNA sequence. They found that—in addition to binding to the gene regulatory regions of *CZF1*, *WOR2*, and *EFG1*—*WOR1* binds to the regulatory region of its own gene and to the regulatory regions of about 60 other genes that are differentially expressed in white and opaque cells, providing more evidence that *WOR1* is a “master regulator” of switching.

The authors present a model of the circuit controlling the white-opaque switch in which *WOR1* directly induces *CZF1* and *WOR2* expression, which in turn activates *WOR1* expression. (*CZF1* does this in a roundabout manner by repressing *EFG1*, which serves as a repressor of white-opaque switching.) The net result is a complex series of positive-feedback loops that give rise to two heritable states. When the feedback loops are inactive, C. albicans assumes the white form; excitation of the loops drive C. albicans into the opaque state. The regulators of this loop are produced at relatively high levels and are presumably inherited by daughter cells, thus ensuring that the loop remains active in progeny cells. A series of interlocking loops (as opposed to a single loop) may buffer the switch against minor fluctuations in the levels of any one component, thereby providing some additional stability to opaque cells as they divide.

The authors observe that this gene-regulatory circuit is similar in principle to the transcriptional feedback loops seen in certain animal developmental processes. That something so complex evolved independently to regulate processes as disparate as eye development in flies, muscle development in mammals, and white-opaque switching in C. albicans suggests that the circuit is an efficient way of using one genome to endow cells with very different properties and of ensuring that these new properties will be stably inherited from generation to generation. The C. albicans white-opaque switch is undoubtedly just one example among many similar circuits that have a large and inherited effect on cells that remain to be discovered.

